# Study on dynamic response and long-term settlement of silty soil around Shanghai metro tunnel

**DOI:** 10.1038/s41598-024-59830-w

**Published:** 2024-04-22

**Authors:** Biaowei Sang, Chunling Yan, Cheng Wang, Xin Qu

**Affiliations:** 1 School of Water Resources and Architectural Engineering, Tarim University, Alear, 843300 Xinjiang China; 2 School of Civil and Architectural Engineering, Anyang Institute of Technology, Anyang, 455000 Henan China

**Keywords:** Metro tunnel, Numerical simulation, Long-term settlement, Metro vibration load, Civil engineering, Computational science

## Abstract

At present, the method for calculating long-term tunnel settlement predictions under metro loading considers only one working condition of passenger loading, which is inconsistent with actual working conditions. To establish a tunnel settlement model that accounts for variations in passenger flow, this study uses data mining methods to categorize metro operation into three working conditions: "peak period, secondary period, and low period." The impact of these passenger flow conditions on the dynamic response of the soil around the tunnel is analyzed. Then, based on the principles of calculus, a calculus-based prediction model is established to consider the changing patterns of metro passenger flow. The model is applied to analyze the long-term settlement characteristics of Shanghai Metro Line 10. The results indicate that, under identical conditions, soil displacement and dynamic deviatoric stress around the tunnel increase with passenger capacity. The calculus prediction model aligns more closely with actual working conditions than the conventional model. The predicted tunnel settlement of Shanghai Metro Line 10 after 20 years of operation is approximately 37.07 mm, with most settlement occurring in the early stages, primarily due to cumulative plastic deformation of the soil.

## Introduction

Shanghai is one of China's mega-cities, responsible for significant economic development and subject to enormous transportation pressure. The metro plays a crucial role in Shanghai's urban transport system, offering advantages such as high speed, large capacity, low pollution, and no occupation of surface space ^1^. Metro operation generates vibrations that affect the soil around the tunnel, which is one of the primary causes of uneven tunnel settlement ^2–4^. Although soil deformation from a single operation may be insignificant, soil deformation accumulates during the tens of thousands of metro load cycles each year ^5^, eventually leading to larger tunnel settlements. For instance, Shanghai Metro Line 1 experienced an average settlement of 111 mm 15 years after opening, with some sections reaching 295 mm ^6^. Shanghai Metro Line 2 saw settlements of up to 170 mm after 11 years of operation ^7^, and Shanghai Metro Line 4 had a maximum cumulative settlement of over 160 mm after 10 years ^8^. Uneven tunnel settlement can impact passenger comfort, increase operation and maintenance costs, and potentially reduce the safety of metro operations. In China's soft soil areas along the southeast coast, the problem of inhomogeneous settlement induced by metro loads is increasingly concerning.

Current prediction methods for long-term tunnel settlement primarily include the field test method ^9^, numerical simulation method ^10,11^, model test method ^12^, machine learning method ^13^, analytical method ^14^, and theoretical research method ^6,15,16^. The focus of these studies is mainly on tunnel structure ^17^, soil type ^18,19^, train speed ^20^, and tunnel surroundings ^15,21^.

Studies on the long-term settlement of metro tunnels under metro loads mostly modify the empirical model of soil's cumulative plastic deformation. They consider only one type of metro loading condition and do not account for the correlation between passenger flow changes and metro load, lacking a long-term settlement calculation method that takes into account the variation in passenger flow. However, Zhang ^22^ found that the amplitude of dynamic stress caused by metro operations is significantly affected by changes in passenger flow, with the amplitude of the dynamic response of the soil around the tunnel during Shanghai Metro Line 1's morning peak hour differing by 15% compared to the noon hour. Therefore, the influence of passenger flow changes on tunnel settlement should be considered in related studies.

In order to develop a tunnel settlement model that takes into account the effects of changes in metro passenger flow, the following steps are taken. Initially, measured passenger flow data are analyzed using the data mining method ^23^, dividing metro train operation into three working conditions: "peak period, secondary period, and low period." A three-dimensional coupling model of the metro-tunnel-soil system is established using Midas GTS NX software to analyze the dynamic response of the metro under each working condition. Then, based on the principles of calculus, an explicit calculus prediction model that considers the three operating conditions is established. The model's strengths and weaknesses are evaluated using Shanghai Metro Line 1 as an example. Finally, the model is applied to analyze the long-term settlement characteristics of the shield tunnel in the silty soil area of Shanghai Metro Line 10.

## Establishment of model

### Model parameters

The tunnel for Shanghai Metro Line 10 has an outer diameter of 6.2 m, an inner diameter of 5.7 m, and is located at a depth of 12.5 m. It traverses soil layers ②_3a_ and ②_3b_, with the physical parameters of these layers presented in Table 1. The tunnel segment is constructed of C55 concrete, while the track bed and floating slab are made of C30 concrete. The soil is simulated using the Mohr–Coulomb model, and the lining, roadbed, and floating slab are modeled using an elastic model. Welded contacts are employed between all model components.

**Table 1:** Table of modelled soil parameters.

Name of the soil	Thickness (m)	Unit weight (kN·m^−2^)	Elastic modulus (MPa)	Poisson ratio	Cohesion (kPa)	Friction angle (ͦ)
① Fill	2	18.0	6.06	0.30	10	10
②_1_ Clayey silt	0.7	18.2	5.10	0.31	10	23.5
②_3a_ Silty silt	6	18.5	7.50	0.30	6	26.5
③_3b_ Silt	6	18.3	6.80	0.32	4	27.5
②_3c_ Silty silt	9	18.6	7.40	0.30	5	27
④ Silty clay	60	17.6	4.30	0.33	15	14.5

**Figure 1:**
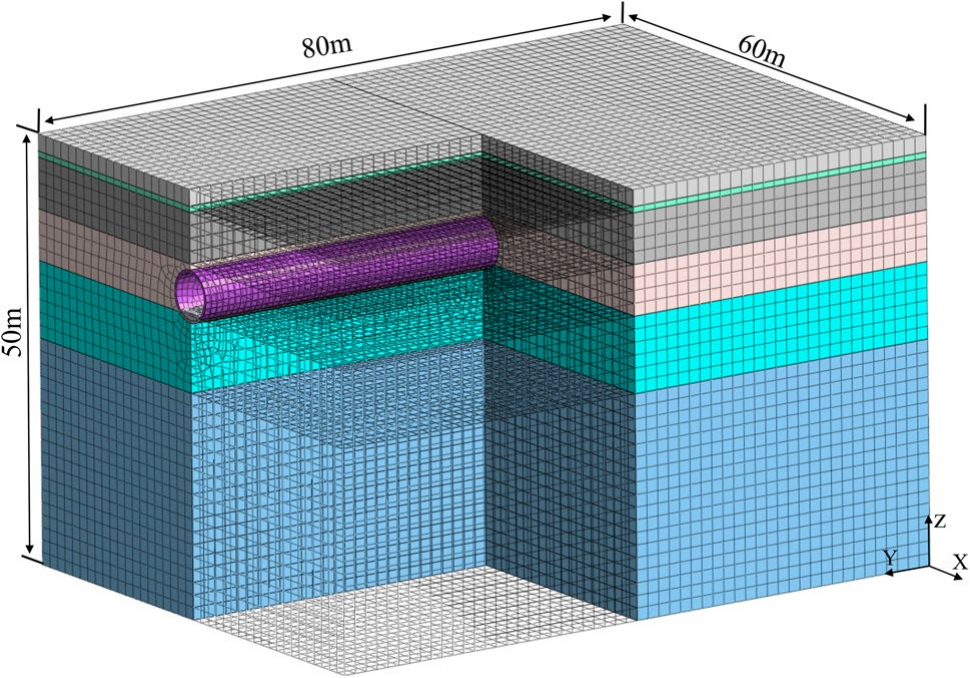
3D numerical model.

Taking into consideration the soil environment surrounding the tunnel and the range of vibration influence ^22^, the transverse width of the model (X) is set at 60 m (equivalent to 5 times the tunnel diameter from the tunnel's center to the outside), the vertical depth (Z) is 50 m, and the longitudinal length (Y) is 80 m. The accuracy of the dynamic analysis model's results is high when the mesh size is smaller than 1/14 of the shear wavelength (2.7 m) ^24^. Thus, the grid size is set between 0.7 and 2 m to balance modelling efficiency, computational accuracy, and computational efficiency. For geostress equilibrium, a statically constrained boundary is used to zero out the displacements. To prevent wave reflection during the application of metro loads, an artificial viscoelastic boundary is utilized. The specific 3D numerical model is depicted in Fig. 1.

### Analysis of metro passenger flow rules

**Figure 2:**
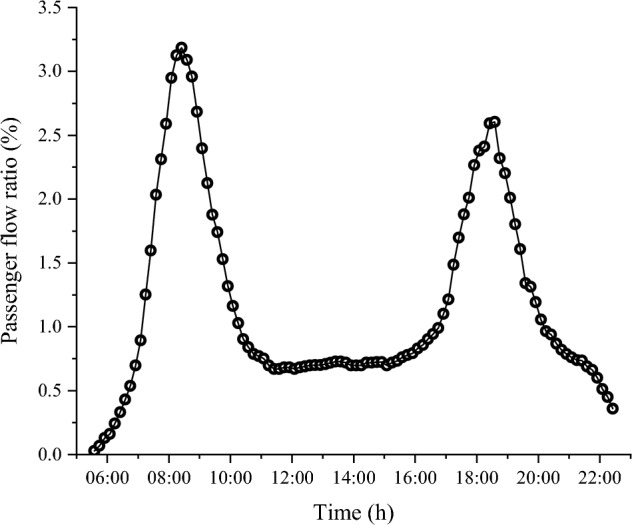
Metro passenger flow curve over time.

The curve illustrating the change inmetro passenger flow is shown in Fig. 2^25^. It is evident from the figure that passenger flow fluctuates significantly within each time period. If only one operating condition were considered in the settlement calculation, the result would not align with the actual operating conditions. Consequently, this study utilizes data mining and the 3–4–5 rule ^23^ to segment metro operations based on passenger volume characteristics across various time periods.

By rounding down the lowest level of metro passenger flow and rounding up the highest level, the metro passenger flow change interval is established as [0,4], which spans four values, centering on the critical number 1, i.e., (4–0)/1 = 4. According to the 3–4–5 rule, this interval can be divided into four equal-width subintervals: [0,1), [1,2), [2,3), and [3, 4]. The number of runs within each subinterval represents 51%, 27%, 16%, and 6% of the total daily runs, respectively. The smaller intervals [2,3) and [3, 4] are combined to create a single interval [2, 4]. Meanwhile, the periods of passenger flow between [0,1), [1,2) and [2, 4] were successively named as low period, secondary period, and peak period.

Upon comparing the number of passengers for each condition, a significant discrepancy is observed. The number of passengers during the peak period is 1.93 times that of the secondary period and 3.92 times that of the low period. Hence, the variations in metro load due to passenger flow changes should be given due consideration. These fluctuations in passenger volume must also be factored into the related settlement calculations.

### Simulation of metro cyclic load

**Figure 3:**
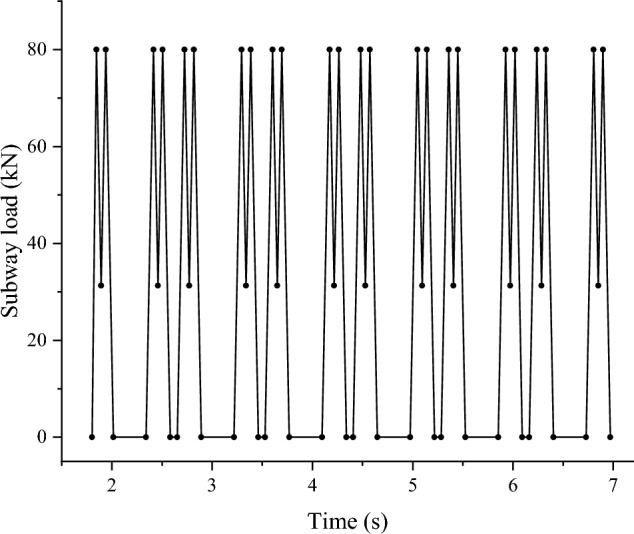
Metro load time chart.

**Figure 4:**
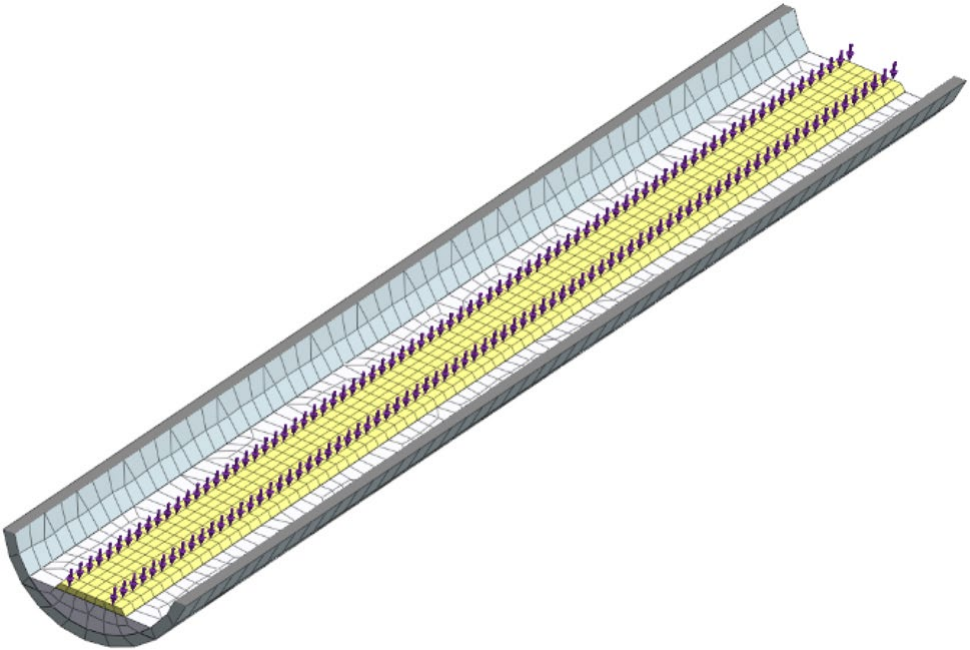
Schematic diagram of metro load application.

Shanghai Metro Line 10 utilizes Type A Metro with a maximum axle weight of 16 tons, capable of accommodating 2,500 passengers at full capacity and traveling at a speed of 80 km/h ^6^. Midas GTS NX software features a metro moving load function that automatically generates the moving load data based on the metro's parameters. The load is set in the model with the metro running from y = 0 m to y = 80 m, treating full capacity as the peak period load condition (with secondary and low periods scaled accordingly). The analysis focuses on the model's central section (y = 40 m), where the metro's dynamic load is depicted in Fig. 3, and the applied load is illustrated in Fig. 4.

## Analysis of model results

The numerical results are analyzed under three metro loading conditions to investigate the variation rule of foundation settlement concerning the vertical and horizontal distance from the tunnel after the first loading, as well as the influence of operating conditions on foundation settlement and dynamic deviatoric stress.

### Tunnel displacement characteristics after the first loading

**Figure 5:**
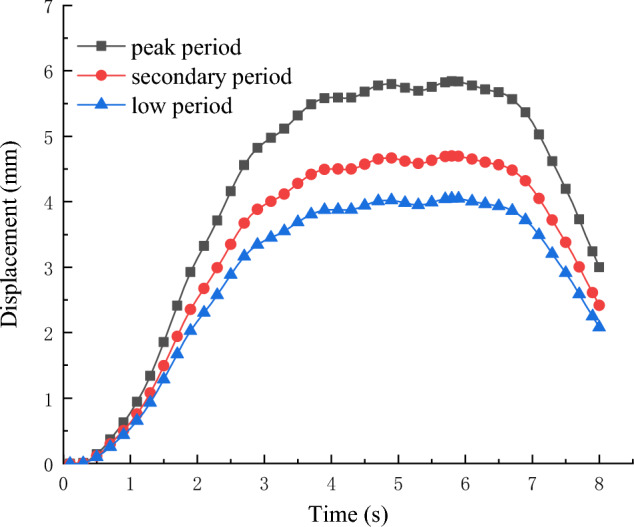
Tunnel vertical displacement time course curve.

Long-term tunnel settlement is primarily related to the soil deformation at the tunnel's base. Taking the soil at the bottom of the tunnel as the observation point, the time curve of the tunnel's vertical displacement for each working condition is depicted in Fig. 5. It is observed from Fig. 5 that the displacement of the soil beneath the tunnel for different working conditions follows a similar period, increasing and then decreasing with the metro's passage. The peak vertical displacement of the soil occurs when the fourth car of the metro runs through the observation section.

The foundation deformation caused by metro vibrations increases with passenger volume. The maximum displacement during the peak, secondary, and low periods is 5.84 mm, 4.71 mm, and 4.05 mm, respectively. As passenger flow decreases from peak to secondary and then to low, the displacement decreases by 19.35% and 30.65%, respectively. It can be concluded that the effect of passenger volume changes cannot be neglected in the study of tunnel settlement.

**Figure 6:**
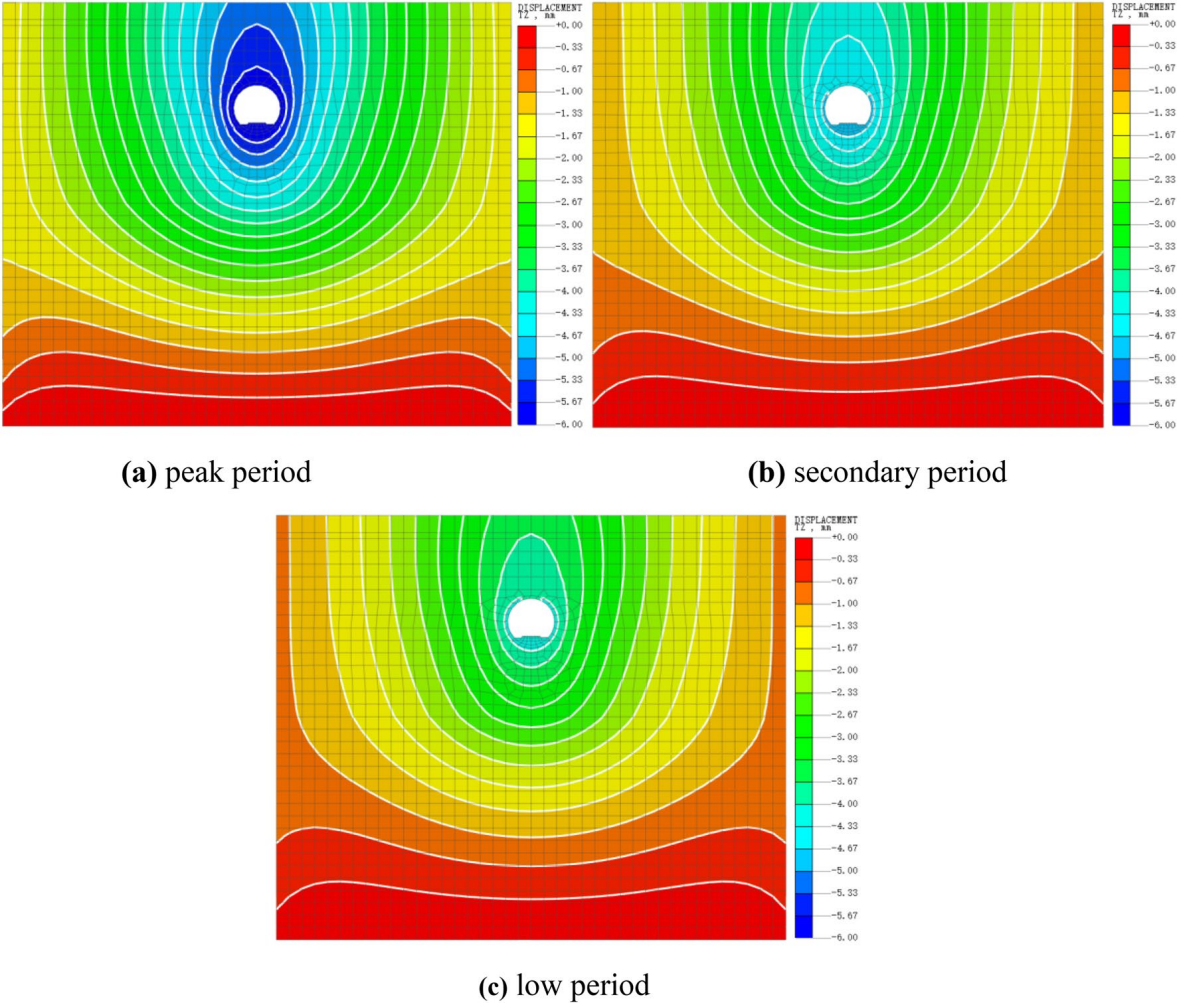
Tunnel vertical displacement cloud image.

The vertical displacements of the three working conditions were plotted as Fig. 6, which reveals the characteristics of displacement development in space. The figure shows that the vertical displacements for the three conditions exhibit the same spatial development trend. Displacement is greatest in the tunnel section and then gradually decreases horizontally outward and vertically downward from the tunnel as the center. This is because the closer the distance to the loaded location, the more the soil is disturbed by external forces, resulting in greater displacement.

### Dynamic deviating stress response rule

The dynamic stress response level of the foundation soil is a critical factor affecting the long-term settlement and deformation of tunnels. The metro-induced dynamic deviatoric stress is typically represented by the second deviatoric stress invariant *q*_*d*_, which can be calculated from the soil stress components using Eq. 1^26^.

1 $$ q_{d} = \sqrt {3J_{2} } = \left\{ \frac{1}{2} \right.\left[ {\left( {\sigma_{xx} - \sigma_{yy} } \right)^{2} + \left( {\sigma_{xx} - \sigma_{zz} } \right)^{2} + } \right.\left. {\left. {\left( {\sigma_{yy} - \sigma_{zz} } \right)^{2} + 6\left( {\tau_{xy}^{2} + \tau_{xz}^{2} + \tau_{yz}^{2} } \right)} \right]} \right\}^{\frac{1}{2}} $$

where: *q*_*d*_ is the dynamic deviatoric stress; *J*_*2*_ is the second stress invariant; *σ*_*xx*_, *σ*_*yy*_, *σ*_*zz*_, *τ*_*xy*_, *τ*_*xz*_, and *τ*_*yz*_ are the stress components under the metro load.

**Figure 7:**
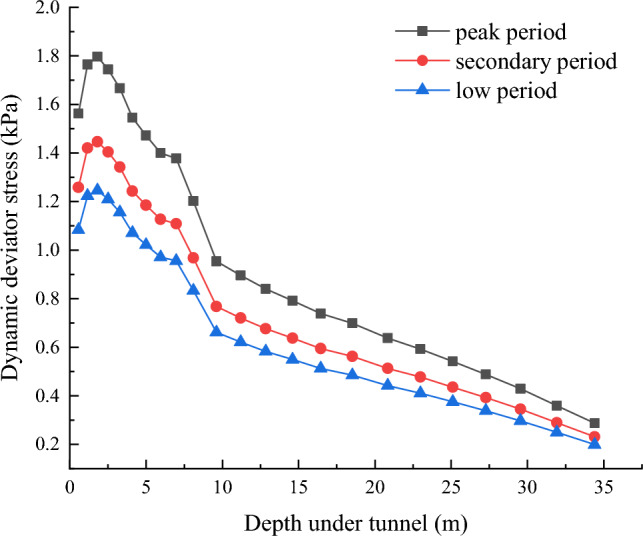
Dynamic deviatoric stress response of the soil underneath the tunnel.

Figure 7 illustrates the dynamic deviatoric stress response curve of the soil beneath the tunnel. It can be seen that the dynamic deviatoric stress initially increases with depth, reaching a peak at 1.8 m, and then gradually decreases. This phenomenon is attributed to the superposition effect of vibrations caused by the different attenuation speeds of the body wave and the Rayleigh wave in the soil layer ^27^, typically occurring 1–2 m below the tunnel ^18,19^. The magnitude and depth of this effect are influenced by factors such as the type of soil layer, surrounding buildings, and tunnel structure. During actual construction, reinforcement measures should be implemented in the vibration amplification area to mitigate the impact of vibration amplification on settlement.

The peak value of the dynamic deviatoric stress decreases as passenger load decreases, with a 19.4% reduction from the peak period to the secondary period, followed by a 13.8% reduction from the secondary period to the low period. Therefore, when calculating the long-term settlement of the tunnel, the influence of passenger loading conditions must be considered to accurately account for the variation in dynamic response due to different operating conditions.

## Calculation model of long-term settlement of tunnel

To account for cumulative plastic deformation under cyclic loading and consolidation settlement due to the dissipation of cumulative pore water pressure, the hierarchical summation method is utilized to calculate the long-term settlement induced by metro vibration.

### Calculation model of undrained cumulative deformation

The cumulative deformation law of a silty soil foundation under metro loading can be expressed by the exponential model in Eq. 2^28^.

2 $$ \varepsilon_{p} = a\left( {\frac{{q_{d} }}{{q_{f} }}} \right)^{m} \left( {1 + \frac{{q_{s} }}{{q_{f} }}} \right)^{t} N^{b} $$

where: *ɛ*_*p*_ is the cumulative plastic deformation; *q*_*s*_ is the static deviatoric stress; *q*_*d*_ is the dynamic deviatoric stress amplitude; *q*_*f*_ is the static destructive deviatoric stress; *N* is the number of cyclic load; *a*, *m*,* t*, and *b* are constants that can be determined through dynamic triaxial testing.

### Calculation model of undrained excess pore pressure

The relationship between excess pore water pressure and the number of load cycles in silty soil under metro loading can be expressed as Eq. 3^29^.

3 $$ \frac{u}{{P_{c} }} = c\left( {\frac{{q_{d} }}{{q_{f} }}} \right)^{g} \left( {1 + \frac{{q_{s} }}{{q_{f} }}} \right)^{k} N^{l} $$

where: *u* is the excess pore water pressure; *q*_*d*_ is the dynamic deviatoric stress; *q*_*s*_ is the static deviatoric stress; *q*_*f*_ is the static destructive deviatoric stress; *p*_*c*_ is the initial effective consolidation pressure of the soil body; *α*, *β*, *γ*, *χ* are the model parameters, which can be derived from the results of dynamic triaxial tests.

### Calculation method of long-term cumulative settlement

The layered summation method is used for calculating tunnel settlement. The specific process is shown in Eqs. 4–Eq. 6^7^.

The settlement *S*_*d*_ caused by the cumulative plastic deformation of the soil under metro loading can be calculated by the Eq. 4.

4 $$ S_{d} = \sum\limits_{r = 1}^{z} {\varepsilon_{pr} H_{r} } $$

where: *ɛ*_*pr*_ the cumulative plastic deformation of the soil in the rth layer; *H*_*r*_ is the thickness of the soil in the rth layer; *z* is the number of soil layers in the compression layer.

The consolidation settlement *S*_*v*_ due to dissipation of cumulative pore water pressure, which can be calculated by the cumulative pore pressure calculation model combined with the Terzaghi one-dimensional consolidation theory by the Eq. 5.

5 $$ S_{v} = \sum\limits_{r = 1}^{z} {mv_{r} H_{r} u_{r} U_{r} } $$

where: *u*_*r*_ the excess pore water pressure of the rth layer; *mv*_*r*_ is the volume compression coefficient of the rth layer of soil; *U*_*r*_ is the degree of consolidation of the rth layer of soil, taking 100%.

The total sedimentation S_*Q*_ is as the Eq. 6.

6 $$ S_{Q} = S_{d} + S_{v} $$

### calculus prediction model

In the long-term operation of the metro, the variation in dynamic response between different working conditions cannot be ignored. To comprehensively consider the influence of each working condition, the conventional model of long-term settlement of underground tunnels is improved by incorporating the principles of calculus. Thus, a calculus-based model that accounts for changes in passenger flow is established.

Assume the number of metro runs per day is *A*, with *i* runs during the peak period, *j* runs during the secondary period, and *k* runs during the low period. Integrate the number of load cycles *N* in Eq. 2 to divide the settlement caused by the first day of operation into three parts. As shown in Eq. 7.

7 $$ \varepsilon_{p1Q} = \int_{1}^{Ah} {d\varepsilon_{p} dN} = \varepsilon_{p1I} + \varepsilon_{p1J} + \varepsilon_{p1K} $$

where: *h* times is the number of loading times per vehicle, *h* = 12 ^21^; *ɛ*_*p1I*_, *ɛ*_*p1J*_, *ɛ*_*p1K*_ are the cumulative plastic deformations of peak, secondary, and peak period conditions in a day, respectively, which can be calculated by the Eqs. 8–10.

8 $$ \varepsilon_{p1I} = \int_{1}^{ih} {d\varepsilon_{p} dN} $$

9 $$ \varepsilon_{p1J} = \int_{ih + 1}^{{\left( {i + j} \right)h}} {d\varepsilon_{p} dN} $$

10 $$ \varepsilon_{p1K} = \int_{{\left( {i + j} \right)h + 1}}^{{\left( {i + j + k} \right)h}} {d\varepsilon_{p} dN} $$

The total deformation after nth day is *ɛ*_*pnQ*_, which can be expressed by Eq. 11.

11 $$ \varepsilon_{pnQ} = \int_{1}^{nAh} {d\varepsilon_{p} dN} = \varepsilon_{pnIQ} + \varepsilon_{pnJQ} + \varepsilon_{pnKQ} = \varepsilon_{p1Q} + \varepsilon_{p2Q} + \varepsilon_{p3Q} + ... + \varepsilon_{pnQ} $$

where: *ɛ*_*pnIQ*_, *ɛ*_*pnJQ*_, *ɛ*_*pnKQ*_ are the total amount of cumulative plastic deformation for peak, secondary, and peak period conditions after n days, respectively, which can be derived from the Eq. 12 ~ Eq. 14.

12 $$ \varepsilon_{{pnI{\text{Q}}}} = \sum\limits_{l = 1}^{n} {\varepsilon_{plI} } = \int_{1}^{ih} {d\varepsilon_{p1} dN} + \int_{Ah + 1}^{Ah + ih} {d\varepsilon_{p2} dN} + ... + \int_{(n - 1)Ah + 1}^{(n - 1)Ah + ih} {d\varepsilon_{pn} dN} $$

13 $$ \varepsilon_{{pnJ{\text{Q}}}} = \sum\limits_{l = 1}^{n} {\varepsilon_{plJ} } = \int_{ih + 1}^{{\left( {i + jh} \right)}} {d\varepsilon_{p1} dN} + \int_{Ah + ih + 1}^{{Ah + \left( {i + j} \right)h}} {d\varepsilon_{p2} dN} + ... + \int_{(n - 1)Ah + ih + 1}^{{(n - 1)Ah + \left( {i + j} \right)h}} {d\varepsilon_{pn} dN} $$

14 $$ \varepsilon_{{pnK{\text{Q}}}} = \sum\limits_{l = 1}^{n} {\varepsilon_{plK} = \int_{{\left( {i + j} \right)h + 1}}^{{\left( {i + j + k} \right)h}} {d\varepsilon_{p1} dN} } + \int_{{Ah + \left( {i + j} \right)h + 1}}^{{2A{\text{h}}}} {d\varepsilon_{p2} dN} + ... + \int_{{(n - 1)Ah + \left( {i + j} \right)h + 1}}^{nAh} {d\varepsilon_{{p{\text{n}}}} dN} $$

Likewise, the cumulative excess pore pressure after n days, considering the three operating conditions, can be calculated by Eq. 15.

15 $$ u_{nQ} = \int_{1}^{nAh} {dudN} = u_{nIQ} + u_{nJQ} + u_{nKQ} $$

In summary, the improved calculus prediction model can be formulated as Eq. 16.

16 $$ S_{{n{\text{Q}}}} = S_{nd} + S_{nv} = \sum\limits_{r = 1}^{z} {mv_{r} H_{r} u_{nrQ} U_{r} } + \sum\limits_{r = 1}^{z} {\varepsilon_{pnrQ} H_{r} } $$

where: *S*_*nQ*_, *S*_*nd*_, and *S*_*nv*_ are the total settlement, cumulative plastic deformation settlement, and consolidation settlement with dissipation of excess pore pressure after day n, respectively. *ɛ*_*pnrQ*_ is the cumulative plastic deformation of the rth layer of soil after day n; *u*_*nrQ*_ is the excess pore water pressure in the rth layer after day n.

### Reliability verification of calculus model

**Figure 8:**
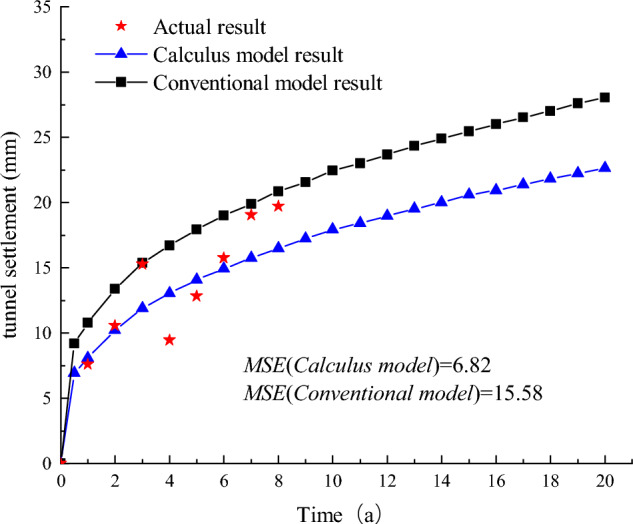
Shanghai Metro Line 1 Settlement.

Figure 8 displays the predicted results of the tunnel settlement for Shanghai Metro Line 1, utilizing both the conventional model ^30^ and the improved model. The Mean Square Error (MSE) of the calculus model is observed to be 6.82, which is lower than the MSE of the conventional model at 15.82. Consequently, the improved calculus model provides a more accurate prediction of the long-term settlement of metro tunnels compared to the conventional model.

### Long-term settlement analysis of Shanghai Metro Line 10 tunnel

**Figure 9:**
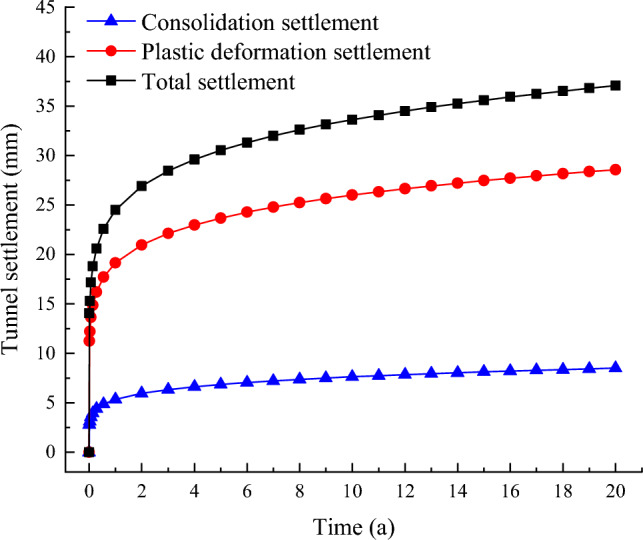
Shanghai Metro Line 10 Tunnel Settlement.

**Figure 10:**
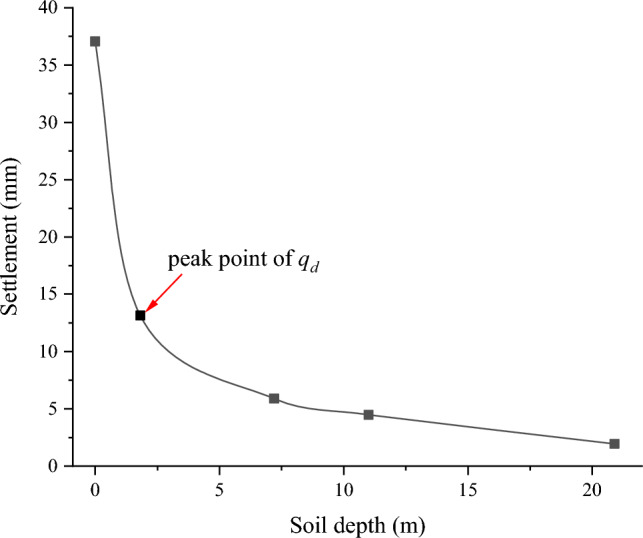
Long-term settlement at depth.

Dynamic triaxial test results from the silty soil surrounding the Shanghai Metro Line 10 tunnel, as reported in the literature [31], yield the following parameter values for Eqs. (2) and (3): *a* = 0.11, *m* = 1.42, *t* = 1.07, *b* = 0.19, *c* = 0.79, *g* = 1.45,* k* = 0.94, and *l* = 0.17. The first metro on Shanghai Metro Line 10 begins operation at 5:25 AM, and the last metro is at 10:30 PM, with metros running at approximately 5-min intervals. Utilizing the improved calculus prediction model, the long-term settlement 20a after the railway's opening has been forecasted, with the results illustrated in Figs. 9 and 10.

As depicted in Fig. 9, the settlements of the Shanghai Metro Line 10 tunnel after 1, 5, 10, and 20a of operation are 24.49 mm, 30.53 mm, 33.63 mm, and 37.07 mm, respectively. It is evident that the long-term settlement of tunnels in a silty soil layer, induced by metro train operations, increases over time. However, the majority of the settlement occurs within the first five years following the commencement of metro services, contributing to 82% of the total settlement. After this period, the rate of settlement decelerates and tends to stabilize. The settlement attributable to the cumulative plastic deformation of the soil amounts to 28.57 mm, representing 77% of the total settlement. Consequently, in relevant projects, it is crucial to prioritize disaster prevention and control related to tunnel settlement during the initial years of metro operation, with a particular focus on the cumulative plastic deformation of the soil.

After two decades of subterranean operation, the settlement of the soil at different depths beneath the tunnel is exhibited in Fig. 10. The maximum settlement displacement, occurring at a depth of 1.81 m (the peak point of dynamic deviatoric stress) beneath the tunnel, is 13.14 mm. This indicates that the long-term settlement of the soil in the 0 to 1.81 m range constitutes 23.93 mm, accounting for 64.55% of the total settlement. Therefore, the deformation of soil due to tunnel settlement predominantly takes place within the 1.81 m depth below the tunnel, specifically in the area where dynamic deviatoric stress vibration is amplified. In relevant projects, reinforcement measures should be concentrated at this depth to mitigate the detrimental effects of differential settlement on the tunnel structure and its operation.

## Conclusions

In this study, the effect of changes in metro passenger flow on the long-term settlement of tunnels was considered. The operation of the underground is divided into three working conditions. Based on this classification, a three-dimensional numerical model of the metro-tunnel-strata interaction is established to compare and analyze the dynamic stress response under these conditions. Subsequently, drawing on calculus principles, an explicit calculus prediction model is formulated, which incorporates changes in passenger flow and investigates the settlement development law of tunnels in silty soil. The main conclusions are as follows:

1. The data mining method is utilized to categorize metro operations into three working conditions: peak, secondary, and low peak periods. Significant differences in passenger load among these conditions are identified. In related projects, it is important to account for the variations in metro load due to passenger flow fluctuations and to consider the impact of these changes in related research.
2. The soil displacement around the tunnel due to metro operations decreases with increasing distance from the tunnel. The dynamic deviatoric stress shows an amplification zone below the tunnel, where the stress increases with depth, peaks at 1.81 m, and then decreases gradually. The vertical displacement and dynamic deviatoric stress response of the soil surrounding the tunnel are exacerbated as passenger capacity increases.
3. The explicit long-term settlement model for metro tunnels is refined using calculus principles, and a calculus model that accounts for the three working conditions is developed. The reliability of this calculus model is confirmed using the measured data from Shanghai Metro Line 1. The results indicate that the improved calculus prediction model is more appropriate for studying long-term tunnel settlement.
4. The total tunnel settlement for Shanghai Metro Line 10 after 20 years is 37.07 mm, with 82% occurring within the first 5 years of the metro's operation. The settlement resulting from the cumulative plastic deformation of the foundation soil constitutes 77% of the total settlement, which is the primary component of long-term tunnel settlement. Tunnel settlement is predominantly caused by soil deformation in the vibration amplification zone, with settlement from this zone accounting for 64.55% of the total. In related projects, it is essential to implement reinforcement measures within the vibration amplification zone. Attention should be directed toward disaster prevention and control of tunnel settlement during the metro's preoperational period, with a particular focus on the cumulative plastic deformation of the soil to prevent uneven settlement from adversely affecting the tunnel's structure and operation.

This study builds on existing research on the prediction method for long-term tunnel settlement by incorporating the impact of passenger flow changes, establishing a settlement calculation model that considers these changes. However, it is important to note that the calculation only takes into account the static load from the structure itself and the effects of metro loads, and that tunnel settlement due to metro operation represents only a part of the total long-term settlement. In the long-term operation of the metro, factors such as construction activity around the tunnel, soil rheology, and changes in the water table will also impact tunnel settlement, possibly leading to deviations between predicted and actual settlements. Therefore, a comprehensive calculation method for tunnel settlement that considers various factors still requires in-depth study.

## Data Availability

The data that support the finding of this study are available from the corresponding author upon reasonable request.

## References

[1] China Association of Metros (2023). 2022 statistics and analysis report of urban rail transit. China Metros..

[2] Tang YQ, Zhang X, Zhao SK, Wang JX, Zhou NQ (2007). Model of pore water pressure development in saturated soft clay around a subway tunnel under vibration load. Chin. Civ. Eng. J..

[3] Zhou PY, Wang JB, Song ZP, Cao ZL, Pei ZM (2022). Construction method optimization for transfer section between cross passage and main tunnel of metro station. Front. Earth Sci..

[4] Ng CWW, Liu GB, Li Q (2013). Investigation of the long-term tunnel settlement mechanisms of the first metro line in Shanghai. Can. Geotech. J..

[5] Yan CL, Tang YQ (2011). Advances in researches on dynamic properties of silty soil under subway loading. Northwestern Selsmol. J..

[6] Shen SL, Wu HN, Cui YJ, Yin ZY (2014). Long-term settlement behaviour of metro tunnels in the soft deposits of Shanghai. Tunn. Undergr. Space Technol..

[7] Wu HN, Shen SL, Chai JC, Zhang DM, Xu YS (2015). Evaluation of train-load-induced settlement in metro tunnels. Proc Inst. Civ. Eng. Geotech. Eng..

[8] Shi YJ, Li MG, Wu W, Wang JH (2018). Analysis of characteristics of long-term longitudinal deformation of tunnel on Shanghai Metro Line No. 4 and its safety evaluation. Tunnel Construct..

[9] Liu JZ, Yang YX, Yang CJ (2021). Analysis and prediction of long-term settlement of metro shield tunnel in saturated sand. Geotech Geol Eng..

[10] Yang Q, Zhang DL, Liu ZC (2012). Numerical simulations of longitudinal settlement of shield tunnel under local loading. J. Beijing Univ. Technol..

[11] Varandas JN, Paixão A, Fortunato E, Zuada CB, Hölscher P (2020). Long-term deformation of railway tracks considering train-track interaction and non-linear resilient behaviour of aggregates -a 3D FEM implementation. Computers and Geotechnics..

[12] Ma XF, Yu L, Li XH (2010). Centrifuge model ling of longitudinal long—term settlement of shield tunnels over lying transitional ground. Chin. J. Underground Space Eng..

[13] Zhang DM, Guo XY, Shen YM, Zhou WD, Chen XS (2024). Data- and experience-driven neural networks for long-term settlement prediction of tunnel. Tunnell Underground Space Technol..

[14] Liu JM (2020). Long-term settlement calculation of shield tunnel in section of deep soft ground in Nansha district Guangzhou. Tunnel Construct..

[15] Huang Q, Huang HW, Ye B, Zhang DM, Gu LL, Zhang F (2017). Dynamic response and long-term settlement of a metro tunnel in saturated clay due to moving train load. Soils Found..

[16] Cai Y, Chen Y, Cao Z, Ren C (2018). A combined method to predict the long-term settlements of roads on soft soil under cyclic traffic loadings. Acta Geotech..

[17] Peng HX, Wang HD, Yuan YH, Li QR (2021). Long-term settlement and control analysis of four-hole overlapping metro tunnel under train cyclic load. Urban Mass Transit..

[18] Wang T, Shi B, Ma LX, Zhang C, Deng YF (2020). Dynamic response and long-term cumulative deformation of silty sand stratum induced by metro train. J. Eng. Geol..

[19] Ma LX, Jin YF, Zhang C, Wang L (2022). Long-term settlements of composite stratum of clay and silt and metro tunnel in it due to train operation. J. Southwest Jiaotong Univ..

[20] Lei HY, Zhang L, Xu YG, Ba ZN (2019). Numerical simulation of settlement of soft soil foundation under fast metro train loads. Chin. J. Geotech. Eng..

[21] Yi HY, Qi TY, Qian WQ, Lei B, Pu BR, Yu YY, Liu YX, Li ZY (2019). Influence of long-term dynamic load induced by high-speed trains on the accumulative deformation of shallow buried tunnel linings. Tunnell. Underground Space Technol..

[22] Zhang X, Tang YQ, Zhou NQ, Wang JX, Zhao SK (2007). Dynamic response of saturated soft clay around a subway tunnel under vibration bad. China Civ. Eng. J..

[23] Han JW, Pei J, Pei J (2012). Data mining: Concept and technology.

[24] Cheng, B. Study on the Displacement Law of Metro Tunnel under Vehicular Vibration. *TongJi University*. (2003).

[25] Luan, Y. Passenger Flow Analysis of urban rail transit under network operation and passenger flow forecast of new lines. *Beijing Jiaotong University*. (2022).

[26] Wang R, Hu ZQ, Ma JK, Li FT, Zhang F (2021). Dynamic response and long-term settlement of a compacted loess embankment under moving train loading. KSCE J. Civ. Eng..

[27] Ma M, Liu WN, Wang WB (2013). Analysis on the reasons of groung vibration amplification induced by railway traffic. Eng. Mech...

[28] Chai JC, Miura N (2002). Traffic-load-induced permanent deformation of road on soft subsoil. J. Geotech. Geoenviron. Eng..

[29] Huang, K. Long Term Cumulative Deformation of silt induced by repeated loading. *Changsha University of Science and Technology*. (2009).

[30] Liu YH, Xu GY, Li W (2022). Study on long-term settlement laws of foundation soil under small-spaced double tunnels. Railway Standard Design..

[31] Yan CL, Hou XB, Chen DG, Zhang JL (2017). Influencing factors of the deformation on the silty soil around the tunnel under subway loading. J. Railway Sci. Eng..

